# Effects of neighborhood streetscape on the single-family housing price: Focusing on nonlinear and interaction effects using interpretable machine learning

**DOI:** 10.1371/journal.pone.0323495

**Published:** 2025-05-21

**Authors:** Jaewon Han, Ayoung Woo, Sugie Lee

**Affiliations:** 1 Department of Urban Planning & Engineering, Hanyang University, Seoul, South Korea; 2 Graduate School of Urban Studies, Hanyang University, Seoul, South Korea; Khalifa University, UNITED ARAB EMIRATES

## Abstract

Previous studies using the conventional Hedonic Price Model to predict existing housing prices may have limitations in addressing the relationship between house prices and streetscapes as visually perceived at the human eye level, due to the constraints of streetscape estimations. Therefore, in this study, we analyzed the relationship between streetscapes visually perceived at eye level and single-family home prices in Seoul, Korea, using computer vision technology and machine learning algorithms. We used transaction data for 13,776 single-family housing sales between 2017 and 2019. To measure visually perceived streetscapes, this study used the Deeplab V3 + deep-learning model with 233,106 Google Street View panoramic images. Then, the best machine-learning model was selected by comparing the explanatory powers of the hedonic price model and all alternative machine-learning models. According to the results, the Gradient Boost model, a representative ensemble machine learning model, performed better than XGBoost, Random Forest, and Linear Regression models in predicting single-family house prices. In addition, this study used an interpretable machine learning model of the SHAP method to identify key features that affect single-family home price prediction. This solves the “black box” problem of machine learning models. Finally, by analyzing the nonlinear relationship and interaction effects between perceived streetscape characteristics and house prices, we easily and quickly identified the relationship between variables the hedonic price model partially considers.

## 1. Introduction

The estimation of housing prices has been a longstanding topic for urban planners and policymakers, as housing units are one of the most expensive commodities in household finances. Housing prices are closely related to macroeconomic conditions, awareness of the streetscape environment, individual preference, and income level [1,2]. In addition, a high-quality pedestrian environment positively affects housing prices [3]. The experience of walking in an environment is directly related to the built environment [4]. In particular, pedestrian environments perceived through walking can be reflected in housing prices as individuals integrate their experiences and behaviors [5,6].

Because most humans acquire information about the external environment primarily through their eyes [7], it is necessary to understand the relationship between a urban’s visual environment and people’s perceptions. However, people’s perceptions of an urban environment are subjective and abstract, which creates technical difficulties in formulating an empirical relationship between an urban environment and perception. Likewise, understanding the experiential relationship between an urban environment as it is perceived in daily life and housing prices is technically difficult. As a result, most studies on this topic have focused on small geographical areas or comparisons of similar urban environments.

The rapid development of computer vision and big data-related technologies is allowing analysis of the large amounts of data available on urban planning. In particular, these technologies can provide big spatial data by precisely measuring the urban environment at the micro-scale and rapidly processing the resulting large datasets to extract detailed trends and patterns of environments, as well as analyzing correlations among data points. Therefore, by utilizing big data technology and machine-learning algorithms, it is possible to identify the nexus between people’s perceptions of the urban environment and socioeconomic characteristics, including housing prices, more accurately than ever before [1,8].

Over the past decade, studies of housing prices have demonstrated how to integrate street-view imagery into hedonic price models to estimate marginal prices for visual characteristics of streets perceived at microscopic scales. For example, Qiu et al. (2022) and Qiu et al. (2023) argue that an objective approach to quantifying microscopic-level perception from pixel information in streetscape images can capture human subjective and subtle sensory processes [6,9]. This means that big data technology and machine learning algorithms effectively complement people’s subjective sensory processes regarding the environment.

Therefore, this study aims to capture the influence of neighborhood streetscape on single-family home prices by integrating street view image big data and computer vision technology into a hedonic price model. To this end, we use interpretable machine learning techniques to solve machine learning algorithms’ “black box” problem and explain the house price prediction results. We also clarify the nonlinear and interactive relationship between neighborhood streetscape characteristics perceived at a human scale and housing prices.

## 2. Literature review

### 2.1. The hedonic price model approach for housing price estimation

Because the factors involved are complex and multidimensional, it is difficult to explain housing prices and the factors that define them [10], which is an obstacle for urban planners and policymakers working to develop the housing market and improve the urban environment [11]. Despite the difficulty, many planners and researchers have discussed relationships between housing prices and factors that affect them.

A representative model is the hedonic price model (HPM) derived from Lancaster’s (1966) [11] consumer theory and Rosen’s (1974) [12] theoretical model. The HPM determines the economic value of heterogeneous goods according to the attributes or characteristics inherent in those goods (Rosen, 1974) [13]. Considering a house as a representative heterogeneous product, housing price prediction studies using the HPM consider the structural properties, location, and environmental characteristics of buildings [6]. The HPM has the advantage of systematically presenting the attribute values inherent in goods based on actual data. However, the HPM-related literature commonly points out some issues in selecting variables and setting and estimating models [14].

The HPM selects independent variables based on empirical research results, theory, and intuitive data. That process often incurs multicollinearity among the independent variables, which reduces the overall reliability of the model [15]. Solving that problem requires omission of variables that cause multicollinearity and collection of additional data [16]. However, that process also raises concerns about model reliability and inefficiency in the analysis because it can cause omission of essential variables or inclusion of irrelevant ones. Furthermore, because the main characteristics of housing prices vary by region, heteroscedasticity in housing prices can occur.

Most importantly, the HPM focuses on estimating the linear relationships between housing prices and other determinants, as the estimation process of the model is fundamentally based on the Ordinary Least Square (OLS) model [17]. These approaches provide the convenience of interpretation and simplicity to identify the relationships between housing prices and various determinants [18]. However, the HPM has limitations in identifying the nexus between housing prices and other factors due to the non-linear relationship inherent in housing properties and prices [19,20]. In addition, the HPM approach is prone to fundamentally violating the statistical assumptions regarding linearity, independence, normality, and equal variance assumed in any OLS estimation, thereby invalidating all such models [21].

### 2.2. Characteristics that determine housing prices

Previous studies that discussed housing price characteristics commonly view the structural, locational, and neighborhoods characteristics of properties as largely affecting neighboring housing prices. When we think of housing as a commodity traded in the market, it is essential that its price is determined by the individual characteristics that make up the house. Additionally, since houses are fixed to the land rather than moving like cars, environmental and locational characteristics can be seen as significantly impacting housing prices [22–25]. Additionally, because housing construction is expensive and tied to the land, the house’s size and the land’s price are the main factors in determining its cost [26–29]. Characteristics related to the size of these homes include the land area, the total floor area of the house, the building coverage ratio, the number of rooms and bathrooms in the home, and the number of floors in the building [30,31].

Since housing is fixed to the land, it interacts with the surrounding environment. In other words, the environmental characteristics, especially regarding the neighborhood and locational attributes of the housing, significantly impact housing prices. Representative examples include parks or green spaces. Green spaces are places for people to rest and provide fresh air simultaneously, contributing to improving the quality of the living environment. Therefore, the more parks or well-maintained green spaces are available around a house, the more likely it is to increase the house price. In addition, as a living environment, the air quality level, safety from crime, excellent walking environment and educational environment are closely related to the living environment surrounding the housing. Therefore, the higher the crime rate [32,33], the worse the air quality [34,35], the worse the walkability [36,37], and the worse the educational environment [38,39], the lower the housing price can be expected to be. In addition, the green spaces, parks, and educational services mentioned above share the meaning of accessibility to some amenities. In other words, people prefer good accessibility to amenities such as commercial facilities, public transportation, and medical facilities such as hospitals, indicating a suitable residential environment. Therefore, a good environment of amenities around a residential area will contribute positively to the increase in housing prices [40,41].

Walking is the most basic transportation when experiencing travel in an urban space. Through this human walking activity, we can experience and interact with various information the city provides. This walking activity is closely related to safety. Recent studies have captured multiple attempts to improve this walking safety and promote walking activity in the city. In particular, studies or models evaluating urban walking performance mainly used three-way and four-way intersections as leading measurement indicators [36,42,43]. In general, four-way intersections can cause more traffic flow and vehicle turns, posing a high risk to pedestrians. Still, in the case of three-way intersections, pedestrians can be relatively safer because the direction of vehicle movement is restricted [44]. In addition, because walking co-occurs with visual experience, many studies have explored the interrelationship between walking activity and urban landscape [8,37].

### 2.3. The effect of the urban landscape on housing prices

A high quality of urban landscape views is an essential consideration for housing buyers [45]. The influence of the urban landscape on real estate prices has been studied for a long time. For example, a higher level of waterfront and open, green views positively affect housing prices [46,47], and many residents prefer natural environments to built ones [48]. Thus, people’s nature-friendly tendencies affect their home-buying behavior [49].

Most daily urban activities occur in residential areas and on the streets. In particular, the streets that constitute a neighborhood environment are physical spaces that induce interaction between humans and the urban environment [50]. Pedestrians perceive their visual environment through frequent head movements while walking and subconsciously assign those visual stimuli with psychological information [51]. For example, tall buildings around streets provide better visual enclosure, and a lower sky view factor is generally perceived as safer [52]. In addition, street trees and greenery provide psychological stability, recovery [53], and aesthetic satisfaction, and safe and well-designed street designs promote pedestrian activity [54]. Therefore, the streetscape as perceived by pedestrians, which considers cities at the micro-scale, determines residents’ psychological satisfaction and behavior, and those factors are reflected in housing prices [6].

Despite many multidimensional studies of the relationship between housing price and landscape, the street environment as it is perceived on a human scale has often been ignored due to data limitations. In addition, many scholars argue that the factors that determine housing prices cannot be fully explained using only structural and environmental characteristics because social experiences, neighborhood awareness, and urban vitality can also affect housing prices [55–57]. Therefore, sophisticated models for housing prices will need to consider landscape attributes as they are perceived on the human scale, the structure of the building for sale, and the characteristics of the neighborhood environment.

### 2.4. Computer vision technology–based streetscape perception at the human scale

Urban landscapes have aesthetic, recreational, and ecological functions for occupants [58], and at the micro-scale, the streetscape experienced by pedestrians can influence human perceptions and behavior [59]. Therefore, it is essential to understand the interaction between streetscape attributes and individuals. Previous studies have analyzed the interaction between landscape attributes and people by subjectively, objectively, and comprehensively measuring streetscape attributes [60]. However, most methods for measuring people’s perceptions of a streetscape depend on qualitative analyses; objective measurements of landscape characteristics have been minimal. In particular, streetscape characteristics at the micro-scale and landscape perception from the pedestrian perspective have been neglected due to a lack of data and methods to analyze them effectively.

Furthermore, because the streetscape can vary depending on the location of the pedestrian and the object being viewed, streetscape data should be measured directly on-site [61]. However, that takes a lot of time and money, so some researchers have used online survey platforms to explore the relationships between landscape attributes and people’s perceptions [62]. However, surveys have limitations in measuring the landscape attributes perceived by pedestrians on a human scale. One recent study focused on quantitatively identifying the elements included in landscape images to address the limitations of qualitative analysis methods [63].

Rapid and accurate data collection and analyses of a large amount of information are possible through advanced computing technology. In particular, convergence of computer vision and big image data technologies makes it possible to measure perceived urban landscape attributes on a human scale. This state-of-the-art methodology can improve understanding about the multiple qualities and values inherent in landscapes [6,56,64,65]. For example, Google Street View (GSV) images are collected from the street and are well suited for measuring urban landscapes as they are perceived from the pedestrian point of view [6,66,67]. In addition, it is possible to use deep-learning technology to understand people’s perceptions of street scenes by extracting and analyzing landscape attributes from many images.

However, these studies have pointed out limitations such as narrow research scope, low-quality data, and the need for more controlled methods to evaluate the impact of visual features on human perception [68,69]. Despite these limitations, methods combining big data and deep learning are increasingly used in urban analysis, and there is a growing focus on model explainability to address concerns about algorithmic bias and transparency. Explainable AI techniques are being applied to improve understanding and prediction of complex phenomena, such as hydro-morphological processes, and to enhance decision-making in risk and risk assessment [70,71].

### 2.5. Issues with previous studies and research gaps

The issues with previous studies that used the HPM to examine the relationship between housing prices and urban landscapes can be summarized as follows. First, the perceived landscape is perceived at a microscopic level, and this sensory process is consistent with the pedestrian perspective. In related studies, landscape features were extracted from pixel information of landscape view images, and the marginal price of the visual features of the landscape was estimated. However, understanding the relationship between perceived landscape and housing prices using only pixel information is insufficient. Second, perception of an urban landscape inevitably differs depending on the viewing location and subject. Therefore, a technology that integrates large-volume data processing and analysis is needed to collect people’s general perceptions of streetscapes. Previous studies lacked the technology required to process and analyze such large-scale data, so people’s general perceptions of streetscapes have not been considered. Third, while the HPM is applicable to estimate and offer results with intuitive interpretation, this model is easily prone to violate the statistical assumptions regarding linearity, independence, normality, and equal variance among variables. However, various factors that affect housing prices tend to not meet these criteria, making the model less reliable. Finally, a detailed approach based on each market’s characteristics is necessary because the housing market is highly segmented. In particular, the housing market can be segmented by housing type, and the streetscape perceived from a pedestrian perspective may be more closely related to single-family houses directly connected to the road.

Therefore, to solve the problems of previous studies and narrow the gap, this study integrates machine learning and deep learning methods into HPM to understand the relationship between neighborhood streetscape and single-family house prices and estimate marginal prices. Additionally, we apply interpretable machine learning methodologies to clarify the nonlinear and interactive relationship between human-scale perceived neighborhood streetscape features and housing prices.

## 3. Methodology

### 3.1. Research scope

**Fig 1:**
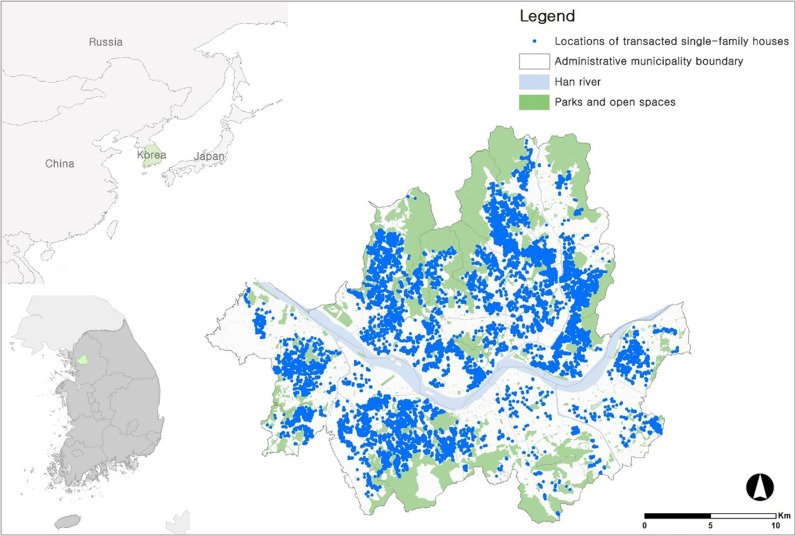
Spatial distributions of transacted single-family houses in Seoul (2017–2019).

The city of Seoul, Korea was chosen for analysis of the relationships between housing prices and external factors. The specific details of the unit of analysis are given in Fig 1.

First, the housing market in Seoul is a barometer for understanding the Korean housing market [72,73]. Therefore, the overall housing market in Korea can be understood through the relationship between housing prices and landscape characteristics in Seoul. Second, most of the urban landscape experienced in daily life occurs from the perspective of pedestrians, and a walkable environment can positively affect landscape perception because it induces people to engage in outdoor activities. Therefore, we paid attention to streetscapes as viewed by pedestrians using GSV images. Third, we focused on single-family houses, which are directly connected to roads and most often to each other and are often experienced through walking.

### 3.2. Data and variables

To determine the nexus between single-family house prices and streetscape factors, we analyzed structural, location, environmental, and landscape characteristics as research data. The dependent variable, housing price, was acquired through a memorandum of understanding with Value Up Systems Co., Ltd. (https://www.valueupmap.com), which operates a land and building trading platform. The data are the actual transaction prices of 13,776 single-family houses sold in Seoul between 2017 and 2019 (Fig 1). Additionally, the research team has received permission from Value Up Systems to collect and analyze the data for academic purposes, not for commercial purposes. Hence, we have identified the source of the data and complied with the conditions outlined in the memorandum of understanding with Value Up Systems Co., Ltd. Meanwhile, we log-transformed the single-family housing transaction prices and input them into the model. This is because we found non-normality when examining the data distribution before analysis, which is related to satisfying the OLS assumption of the hedonic price model.

The analyzed structural characteristics of each building are its site area, gross floor area, age, structure, land topography, site shape, corner parcel, and parking availability, which we analyzed using data from Value Up Systems. For the locational characteristics, we estimated the Euclidean distance from each single-family house to the nearest park and the Central Business District (CBD) boundaries using Seoul Municipal Facilities data from 2018.

The characteristics of walkable environments were measured using macro-scale variables and micro-scale streetscapes. To estimate the characteristics of these walkable environments, the 400m Euclidean distance, the walkable distance from the center point of each single-family house, was used as the spatial unit of analysis [74–76].

**Fig 2:**
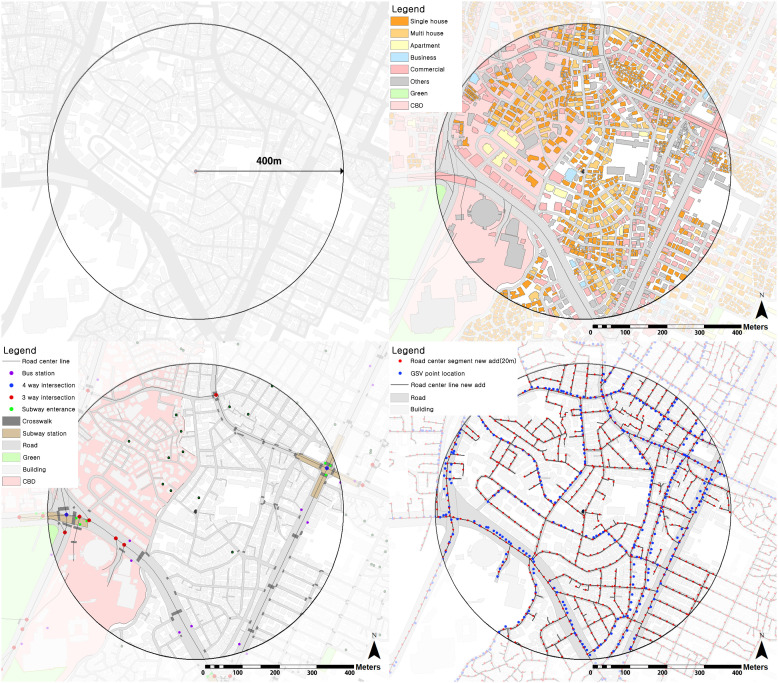
Example measurement and analysis units for landscape elements. Note: This figure shows the method and current status of measuring the built environment within a 400m radius around a single-family house. This includes information on land use, amenities, and the location where the GSV image was taken within a 400m radius of the single-family house.

The density of three-way and four-or-more-way intersections in each walking environment was measured on the macro-scale. To that end, the 2018 Korea Transport Data Base estimated the number of three- or four-or-more-way intersections and the total length of roads in the 400m buffer around each house. In addition, because crosswalks are closely related to pedestrian safety, we used 2018 Seoul Open Data Square (SODS) data to estimate the crosswalk density near each house based on the number of crosswalks and the total length of the road. Using 2018 SODS data and the New Address Database, we estimated the density of bus stops and subway stations around all the single-family houses sold. Furthermore, the amenities of public transportation can be quantified by the density of individual stops. However, in the case of the subway, there is only one station, but there are multiple entrances, so the analysis was conducted using the density variable, which is the subway entrance included in the unit buffer based on the subway entrance. We estimated each neighborhood’s land use characteristics (e.g., residential, commercial, business, and mixed uses) (Fig 2).

Finally, we used deep-learning techniques to estimate the micro-scale streetscapes around single-family houses. The central path of Seoul roads was divided into 20m intervals, and the location coordinates of each segment were extracted using a Geographic Information System (GIS) program. The coordinates extracted in this way were entered into the Application Programming Interface (API) to download the GSV image from each location. Because landscape images can be constituted by different elements depending on season, it is necessary to consider the date and season. Therefore, we assessed the metadata for the street view images provided and analyzed only GSV images from 2018, excluding those from winter (December, January, and February).

**Fig 3:**
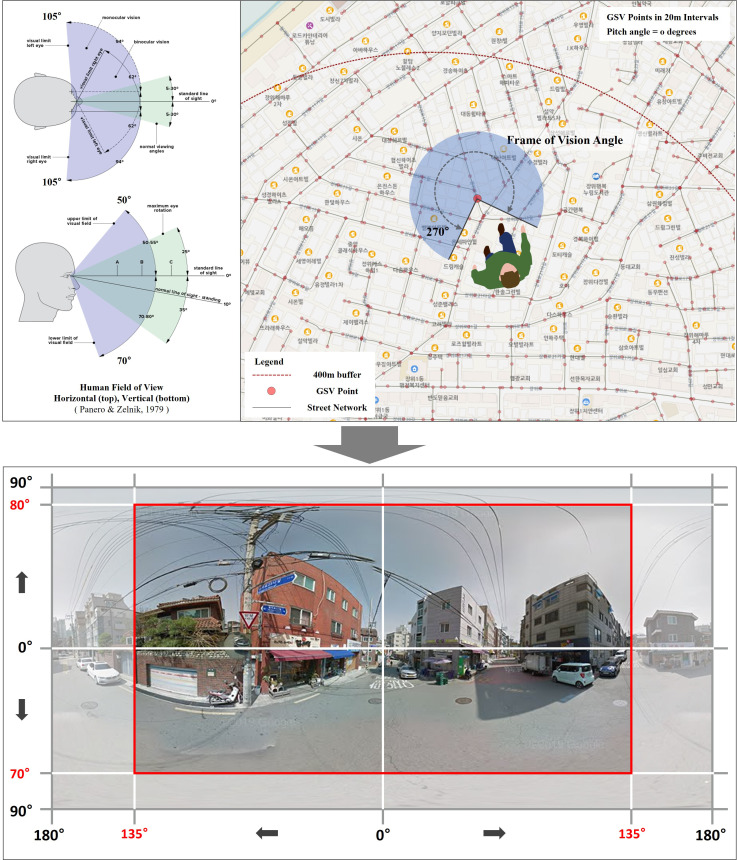
Field of view of the extracted GSV panoramic images.

**Fig 4:**
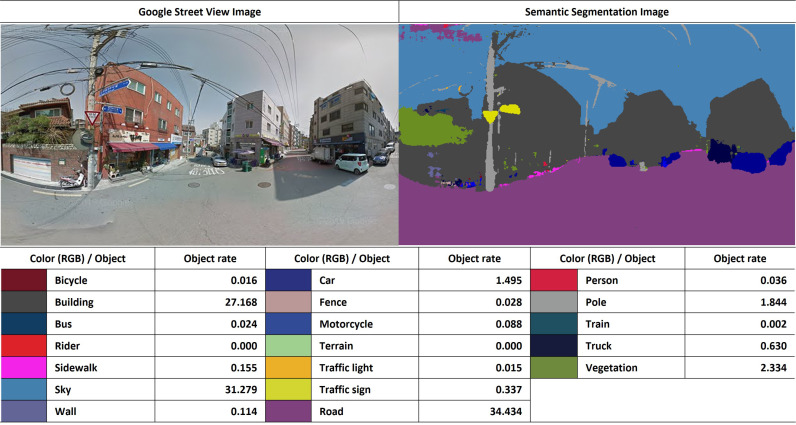
Example of semantic segmentation based on GSV panoramic images.

Because the street view image obtained through the API is panoramic, it differs from the human visual range [77,78]. Therefore, each 360° street view image was cropped to match the range of human vision, and the features in the landscape images were estimated using a deep learning-based semantic segmentation method (Figs 3 and 4). We measured the pixel information of the attributes included in the horizontal view image considering the human field of view. However, there is a limit to inputting the pixel information extracted from the image into the analysis model. This is because the image is 2D information, and the landscape characteristics may be overestimated depending on the shooting location. Therefore, in this study, we recalculated it using a formula and inputted it into the analysis model.

*Greenery* describes the degree of street vegetation visually accessible to pedestrians, and *Enclosure* identifies the proportion of vertical elements, such as buildings, and horizontal features, such as roads and sidewalks. *Paved sidewalks* describe the extent of sidewalks visible to pedestrians. In this study, those landscape attribute values were extracted from individual GSV images included in the 400m buffer around each house, which is the unit of analysis, using a deep learning technique, and the indicators of greenery, enclosure, and paved sidewalks were estimated as averages [46,47]. The average values were calculated using the following formulae.


$$Greenery=\frac{number\;of\;vegetation\;pixels}{total\;pixels}\times 100$$

$$Enclosure=\frac{building\;pixels}{raod\;pixels+sidewalk\;pixels}\times 100$$

$$Paved\;sidewalks=\frac{sidewalk\;pixels}{road\;pixels+sidewalk\;pixels}\times 100$$

### 3.3. Methodology of the machine learning algorithm

In this study, we compare and analyze the predictive power of the random forest, gradient boost, and extreme gradient boost (XGBoost) methods, the most-mentioned machine-learning algorithms, and a linear regression model. The primary metrics used to compare the performances of the models are the coefficient of determination (R^2^), mean squared error, and mean absolute error. High R^2^ values and low mean squared error and mean absolute error values indicate good model performance [65]. Machine-learning algorithms have suffered from a structural problem called the black box, but SHapley Additive exPlanations (SHAP) have recently been proposed to correct that. Therefore, we used SHAP, an interpretable machine-learning method, to predict and interpret single-family house prices.

Despite its advantages, the SHAP method cannot precisely explain non-linear relationships between independent and dependent variables. Therefore, we had to consider additional methods to explain the non-linearity between urban landscape characteristics and housing prices. For this study, we inferred non-linear relationships between the independent and dependent variables using the Partial Dependence Plot (PDP) method.

**Fig 5:**
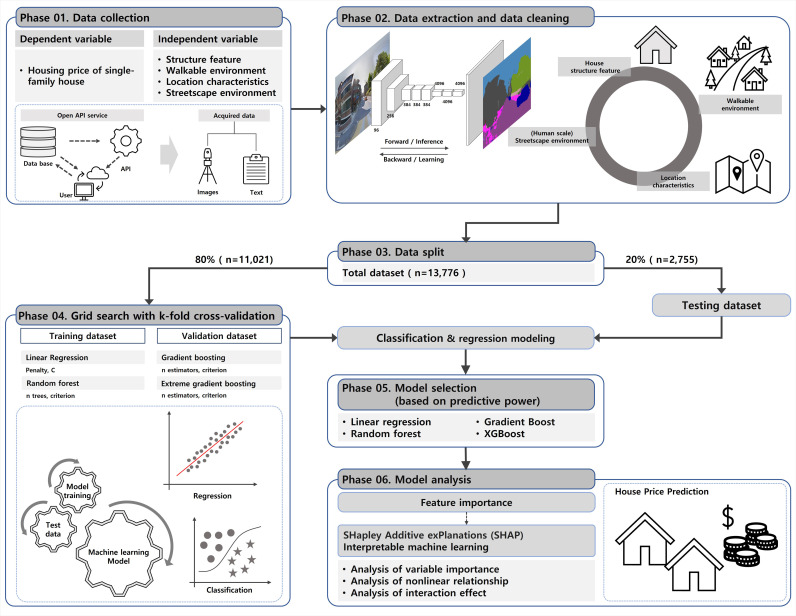
Framework of the machine learning algorithm for single-family housing price.

The process and method of this study are divided into six phases, as shown in Fig 5. In the first phase, actual transaction price data for detached houses were obtained through a memorandum of understanding with Value Up Systems, a big-data property evaluation company, and we collected GSV images and neighborhood environment variables from the API. In the second phase, we extracted the characteristics of each neighborhood environment within a 400m radius (the area of walking activities in daily life) of each housing transaction, and we used deep learning–based semantic segmentation to measure the streetscape variables at the human scale from the GSV images for each 400m buffer area. In the third phase, we divided the data into training, verification, and test sets to establish the machine learning models for housing price prediction. The training and verification data comprised 80% of the data set, and the remaining 20% constituted the test data. In the fourth phase, we used 80% of the total data for training and verification. We used linear regression, random forest, gradient boost model, and XGBoost prediction models and performed hyperparameter tuning to obtain optimal prediction results. The tuning method was a grid-search with k-fold cross-validation. Then, we compared the test data and training data. In the fifth phase, we compared each model’s prediction of single-family house prices and selected the model with the highest predictive power. In the sixth phase, we used interpretable machine learning to analyze the importance of the variables, the non-linear relationships among them, and their mutual effects, focusing on the model with the best predictive power (Fig 5).

## 4. Results

### 4.1. Descriptive analysis

Because housing, as a product, has the characteristics of a substitute with the same utility, consumer perceptions and experiences affect housing prices [79]. In this study, we assumed that the safety, comfort, aesthetics, etc., experienced by pedestrians would significantly influence housing prices and then tested and analyzed that assumption. Meanwhile, housing prices increase as land and floor areas increase, but the growth rate decreases after a critical point [80–83]. This violates the linearity assumption in the hedonic price model. Therefore, for sophisticated modeling, we took the logarithm of the single-family home price and set it as a dependent variable.

First, among the structural characteristics of the houses bought and sold during this study, the average building floor area was 122.63m^2^, larger than the individual family housing size of 85m^2^. Considering that the appropriate residential area per person is 16m^2^, each single-family house’s per capita residential area was bigger than in other housing types for households of the same size. In addition, the site area was 140.33m^2^, which was similar to the average building floor area and indicates that the site area affects the size of the building. The average construction age of the single-family houses sold was 39.7 years, which is higher than the 30-year standard for judging housing deterioration in the Residential Environment Improving Index System adopted by the Seoul Metropolitan Government. Therefore, many single-family houses in Seoul have undergone considerable deterioration. Among the walking environment characteristics around single-family houses, the average value of bus stop density, which is closely related to walkability, was 0.01, indicating that the public transportation conditions around single-family houses are poor, and walkability is low. The average ln values for greenery, enclosure, and paved sidewalks, which are visual recognition variables in the landscape images, were 1.63, 4.69, and 2.52, respectively. The reason ln conversion was applied to the housing price at this time is that the application of rescaling was considered because it was judged that the dependent variable, the housing price, was relatively too large compared to other independent variables. Compared with the average park green-area ratio of about 27.77% within a 400m buffer of housing, the visually recognized green area in the neighborhoods of single-family houses was relatively low (Table 1).

**Table 1:** Definitions of the variables and basic statistics.

Variables	Measurements		Mean	S.D.	Min.	Max.
**Dependent variable**						
ln (housing prices)	The natural logarithm of the price of a single-family house (KRW)		20.17	0.55	17.32	21.24
**Independent variables**						
***Structural features***						
Site area	The site area of a single-family house (m^2^)		140.33	1,875.98	3.00	220,220
Gross floor area	The total floor area of a single-family house (m^2^)		122.63	70.21	4.50	479.94
Housing age	Age of the building		39.70	11.06	0	95
Building structure: Ferroconcrete	Ferroconcrete = 1, others = 0		0.01	0.12	0	1
Building structure: Masonry	Masonry = 1, others = 0		1.46e-03	0.04	0	1
Land topography	Flatland = 1, others = 0		0.67	0.47	0	1
Site shape	Regular form = 1, others = 0		0.84	0.37	0	1
Corner parcel	Corner parcel = 1, others = 0		0.13	0.33	0	1
Parking availability	On-site parking allowed = 1, on-site parking not allowed = 0		0.01	0.12	0	1
***Walking environment***						
***Macro-scale walking environment factors***						
3-way intersection density	(Number of 3-way intersections/total street length) within the 400m buffer		3.29E-03	1.59e-03	0	0.02
4-or-more-way intersection density	(Number of 4-or-more-way intersections/total street length) within the buffer		6.85e-04	5.34e-04	0	3.51e-03
Subway station density	(Number of subway stations/total street length) within the buffer		5.73e-04	9.55e-04	0	0.01
Bus stop density	(Number of bus stops/total street length) within the buffer		0.01	0.05	0	3.56
Crosswalk density	(Number of crosswalks/total street length) within the buffer		0.02	0.06	0	7.34
Park land use	Total park and open space area within the buffer (m^2^)		44,431.24	66,091.75	0	447,553.80
Residential land use	Total area for residential use within the buffer (m^2^)		441,246.40	183,936.50	1,615.14	1,421,861.00
Business land use	Total area for businesses within the buffer (m^2^)		19,325.62	40,873.86	0	917,400.30
Commercial land use	Total area for commercial use within the buffer (m^2^)		73,537.93	54,375.07	0	681,313.20
Mixed land use	$-\sum_{i=1}^{k}\frac{\left(p_{i}\right)*ln(p_{i})}{ln(k)}$	$p_{i}$=Total area of land use in the 400m buffer	0.15	0.02	0.97	0.97
		k=Number of land uses				
***Streetscape environment***						
ln (greenery)	The natural logarithm of the average greenery value within the buffer		1.63	0.46	0.18	3.93
ln (enclosure)	The natural logarithm of the average enclosure value within the buffer		4.69	0.22	1.93	5.14
ln (paved sidewalks)	The natural logarithm of the average paved sidewalk value within the buffer		2.52	0.12	2.01	3.13
***Locational characteristics***						
ln (distance to parks)	The natural logarithm of the distance to the nearest park boundary (m)		4.86	1.00	0	6.95
Distance to CBD	The shortest distance to the CBD boundary (m)		3,765.00	2,242.81	0	11,539.12

### 4.2. Comparison of predictive power of the machine learning models

To compare the predictive power of the machine-learning models, we divided the data for the 13,776 house sales into training data and evaluation data. Machine-learning modeling was performed using 11,021 learning samples, 80% of the total, and 2,755 evaluation points, 20% of the total data.

When we compared the models’ R^2^ values, which indicate their explanatory power, the predictive power values of the linear regression model, XGBoost model, and gradient boost model were 0.660, 0.765, and 0.817, respectively. The two machine learning models outperformed the regression model. The predictive power of the gradient boost model was 81.7%, which is 5.2% better than the 76.5% predictive power of the XGBoost model and 15.7% better than the predictive power of the OLS, a traditional statistical technique. Therefore, the gradient boost model was judged to be the most suitable machine-learning model for housing price prediction (Table 2).

**Table 2:** Performance comparison of machine learning models.

Model	Performance evaluation indicator			
	R^2^	MSE	MAE	MedAE
Linear Regression	0.660	0.104	0.248	0.197
Random Forest	0.318	0.209	0.363	0.318
XGBoost	0.765	0.072	0.200	0.155
Gradient Boost	0.817	0.056	0.169	0.122

The gradient boost model differentiates a loss function generated during modeling with a parameter value that uses a gradient descent method and changes the parameter value to minimize the loss value. In this study, the grid search method was used to derive the parameter values representing the optimal predictive performance, and the adjusted parameter values were subsample = 0.3, n_estimators = 2000, max_depth = 30, and learning_rate = 0.01.

### 4.3. Analysis of variable importance

**Fig 6:**
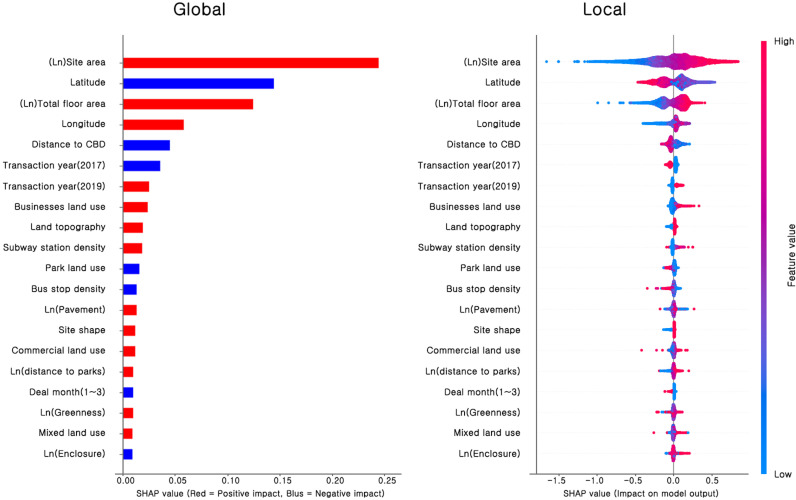
Analysis of variable importance to single-family housing prices using the SHAP method.

Fig 6 shows the global and local Shapley values for the gradient boost model.

In the global Shapley value graph, the variables that increased house prices (positive effect) are shown in red, and those that decreased house prices (negative effect) are shown in blue. The global Shapley value graph represents the average influence of a variable by calculating the absolute Shapley value for each variable. The local Shapley value graph shows data values as points, and a long tail indicates that extreme measurement values affected the prediction results. Mixed red and blue dots indicate the presence of a non-linear relationship between that variable and the predicted value (i.e., single-family house prices). We interpret the global and local Shapley values as follows.

First, the factor with the most significant influence on single-family house prices is site area because the developmental scale of individual buildings and single-family housing prices have a close relationship. Similarly, the total floor area of a building was found to positively affect the predicted price of a single-family house.

Second, latitude (north-south) and longitude (east-west), which represent the location properties of the houses, had negative and positive effects on the predicted price of single-family houses, respectively. The polarization of the housing supply within Seoul and the differences in urban infrastructure are severe, and those differences are reflected in housing prices. In addition, in the local Shapley value graph, the red and blue dots are mixed, which indicates that a non-linear relationship exists between location (latitude and longitude) and single-family house prices.

Third, the distance from the city center and the green area around each single-family house, which also represent location characteristics, had a negative effect on the predicted price of single-family houses. The CBD and parks are perceived negatively in a residential environment because they draw many people from outside the neighborhood. On the other hand, the business district area, commercial district area, and subway station density had positive effects because the various benefits provided by the commercial and business facilities that concentrate around subway stations. In the local Shapley value graph, the red and blue dots are mixed, indicating a non-linear relationship between the location and environment around a neighborhood and single-family house prices within it.

Fourth, bus stops and land use mix, which are closely related to walkability, had negative and positive effects on housing prices, respectively. Many people visit a bus stop, which are often near busy streets and experience various pollutants from vehicles. These characteristics result in a negative perception of the living environment. A higher land use mix, on the other hand, made a residential environment more favorable because it can contain various city functions.

Fifth, the recognized landscape elements (greenery, enclosure, and paved sidewalks) were found to significantly affect single-family house prices. Paved sidewalks and greenery had a positive effect on single-family house prices, and enclosure had a negative effect. The recognized paved sidewalks included both pedestrian paths and roads, and we judged that the perceived pedestrian environment and benefits provided by road infrastructure are reflected in housing prices. Also, abundant greenery in a neighborhood is highly preferred because it offers visual enjoyment and relaxation. Therefore, the perceived green area around a street positively affected single-family house prices. On the other hand, enclosure had a negative relationship with single-family house prices because more people come and go in urbanized areas with denser buildings, which is considered a detriment in a residential environment. Again, the red and blue dots in the local Shapley value graph are mixed, indicating a non-linear relationship between the perceived landscape variables and the price of a single-family house.

### 4.4. Non-linear relationship analysis using PDP

**Fig 7:**
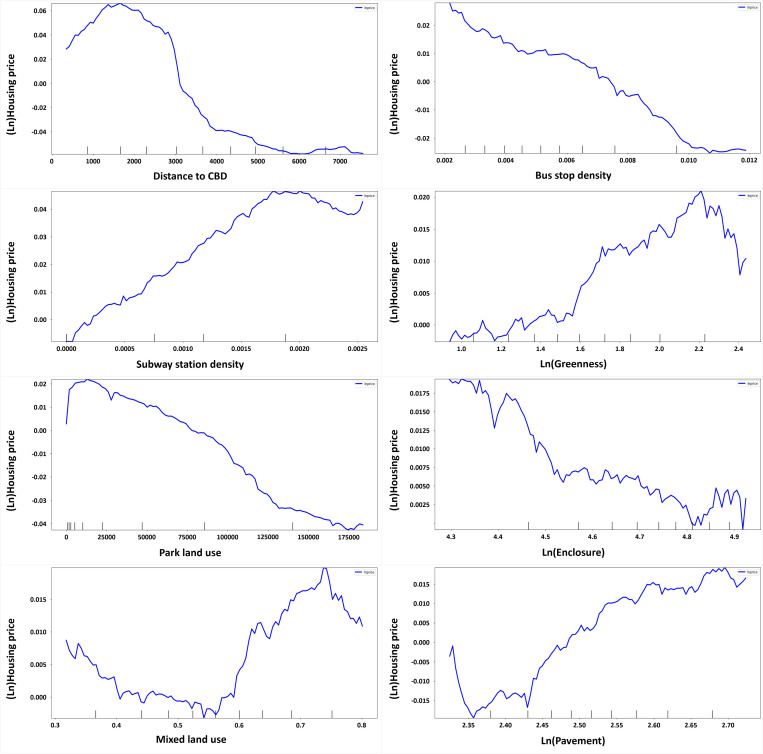
Partial dependence plot analysis of major variables that affect housing prices.

The non-linearity of variables can be confirmed in detail through a PDP (Fig 7). The PDP analysis was performed for the variables with mixed red and blue dots in the local SHAP graph. The X axis represents a factor in predicting single-family house prices, and the Y axis is the single-family house price. According to the local Shapley graph, the neighborhood location environment (distance to CBD, subway station density, and park land use), walkable environment (land use mix and bus stop density), and perceived landscape (greenery, enclosure, and paved sidewalks) variables had a non-linear relationship with housing prices, and PDP analysis was performed for those variables.

Distance to the CBD, subway station density, and park land use are the main factors representing the location environment characteristics of neighborhoods. First, when the distance to the CBD was 1.5 km, its predictive power for single-family house prices was highest, and that power decreased rapidly from that point. In general, the CBD is an urban area with little activity at night and is an unsuitable residential environment. In other words, people’s negative perceptions of the CBD as a living environment are reflected in housing prices. Second, subway stations form influence areas, and the commercial facilities contained in those areas provide various benefits to residents. In the PDP analysis, the predictive power for single-family house prices increased until the density of subway stations reached 0.008 and decreased thereafter. Apparently, the commercial district that forms around subway stations provides various benefits to residents, but beyond a critical level, negative perceptions increase due to concerns about noise, cleanliness, and the numbers of people coming and going. Third, the predictive power of parks on housing prices was highest when the nearby park area was 15,000m^2^, and it decreased rapidly thereafter. Parks are the city’s representative areas that provide citizens with fresh air and a place to rest, as well as habitats for birds and insects. However, parks can be visited frequently by outsiders, which is closely related to the cleanliness of the living environment and safety from crime. Both the positive and negative perceptions of parks are reflected in their influence on housing prices.

In this study, we paid attention to the urban landscape as it is recognized from a pedestrian’s point of view. Walking activity is directly related to the perceived walking environment. A mix of land uses and bus travel are closely related to walking, such that a higher mix of land uses and more bus travel correlate with more frequent walking. When the land use mix was 0.56, its effect on the price predictive value of a single-family house was lowest. Above that, house price predictive value increased rapidly until the land use mix reached 0.72, when it decreased again. A high land use mix allows people’s walking activities to lead to various activities, which can be convenient. However, too many people walking to activities can cause noise and traffic problems, which can be perceived negatively in a living environment. Therefore, the land use mix and housing prices have a non-linear relationship.

In this study, we also studied the relationship between the perceived streetscape and single-family house prices by focusing on the elements of greenery, enclosure, and paved sidewalks. When the greenery value was 2.22, its effect on single-family house price predictive value was highest, and it decreased rapidly from that point. In other words, prosperous greenery around the pedestrian path is deemed to have a positive influence on the living environment, but only to a certain point. Above that level, it is perceived negatively. When the enclosure value was 4.8, its effect on house price predictive value was lowest, and it increased only slightly after that because the enclosure value increases in the presence of more buildings than roads. In other words, as people’s perception of buildings increases, the perception of a negative pedestrian environment is reflected in the housing price, but that effect is not significant above a critical value. When the paved sidewalk value was 2.36, its effect on single-family house price predictive value was lowest. In other words, paved sidewalks can positively affect the price predictive value of single-family houses above a certain level of presence.

### 4.5. Analysis of interaction effects

**Fig 8:**
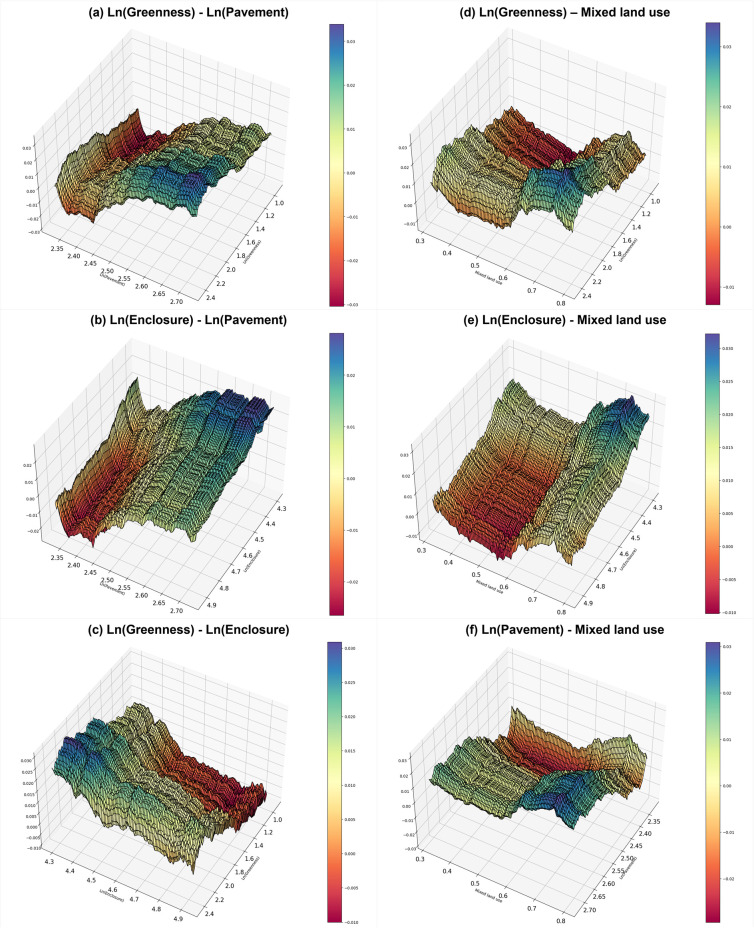
The interaction effects among perceived landscape factors and land use mix.

Land use mix and walking are closely related [84], and the appearance of urban landscapes differ depending on land use characteristics [85]. Furthermore, because the factors that constitute an urban environment are recognized comprehensively, they can collectively affect people’s perceptions and behaviors. Therefore, we analyzed the interaction effects between perceived landscape, land use mix, and predicted single-family housing prices (Fig 8).

Fig 8a shows the correlation effect between the greenery and paved sidewalk values and the price predictive value of a single-family house. When the index value for paved sidewalks was 2.65 to 2.70 and the greenery value was 2.0 to 2.2, the predicted price of a single-family house was high. In other words, the quantity of roads or walking paths and green areas visible during walking, including street trees around roads, can increase the price predictive value of single-family houses.

Fig 8b shows the interaction effect between the enclosure and paved sidewalk values and the price predictive value of a single-family house. When the enclosure value was 4.3–4.4 and the paved sidewalk value was 2.65–2.70, the predicted price of a single-family house was high. In other words, when more sidewalks and more buildings are visible when walking, the positive perception of pedestrian safety can be reflected in housing prices.

Fig 8c shows the effect of the interaction between the greenery and enclosure values on the price of a single-family house. The predicted price of a single-family house was high when the greenery value was 2.0–2.2 and the enclosure value was 4.3–4.4. Therefore, the price predictive values of a single-family house is high when the image ratio of vertical elements (e.g., buildings, street trees) to horizontal elements (e.g., roads, sidewalks) is low and the greenery ratio is above a certain level. In other words, an excessive concentration of buildings causes negative perceptions due to expectations of noise, a poor living environment (e.g., ventilation, lighting, pollution, etc.), and frequent vehicle traffic. But buildings and trees around the street also provide visual enjoyment and relaxation, which can generate positive perceptions among residents.

Fig 8d shows the effect of the interaction between the greenery value and land use mix on the price of a single-family house. Single-family house price predictive values were high when the land use mix index was 0.7 to 0.8 and the greenery value was 2.0 to 2.2, which suggests that land use mix can promote walking activities, which expose people to green landscapes.

Fig 8e shows the interaction effect of enclosure value and land use mix on the price of a single-family house. Single-family house price predictive values were high when the land use mix index was 0.7–0.8 and the enclosure value was 4.3–4.4. Enclosure represents the ratio of vertical elements (e.g., buildings., etc.) to horizontal elements (e.g., sidewalks, roads., etc.), and a higher land use mix correlates with high-rise and high-density development, which increases the ratio of buildings to roads. Thus, a high land use mix can increase the sense of enclosure from a pedestrian’s point of view.

Fig 8f shows the interaction effect between the paved sidewalk value and the land use mix on the price of a single-family house. When the land use mix was 0.7 to 0.8 and the paved sidewalk value was 2.65 to 2.70, the predicted price of a single-family house was high. A high percentage of footpaths with a high land use mix is closely related to walkability. Therefore, land use mix can interact with walkability in ways that are reflected in housing prices.

## 5. Discussion

By examining visual attribute patterns within streetscapes, we can derive insights that aid in devising strategies to optimize housing price prediction. This topic is of paramount importance to urban planning and design, as it facilitates the creation of effective strategies to improve the neighborhood’s visual environment and amplify the potential of providing various housing types in urban settings. To contextualize our findings within the broader spectrum of housing price predictive and characteristic factors analysis, it is imperative to consider and leverage relevant studies conducted by other researchers to contextualize our findings within the broader spectrum of housing price prediction analysis.

For example, Yang et al., Woo et al., and Lin et al. [8,56,69,86–89] developed a model to explore the relationship between streetscape features variables and housing price. Similarly, Better Street design is closely related to housing price increases [6], and many studies have used the HPM to analyze the effects of urban landscape features on housing prices. Landscape parameters that affect housing prices are theoretically challenging to measure and approach, often have complex characteristics, and do not satisfy an exact linear relationship due to spatial correlations between independent variables [10,90,91]. Because they violate linearity, which is a structural assumption of the HPM, model establishment is denied. In contrast, our study primarily relies on the pedestrian view and explainable AI to predict housing prices. It is also noteworthy that many preceding studies in this domain are data-constrained, often focusing on specific housing markets or housing types [39,92].

In this study, we assumed that the perceived urban landscape would correlate with housing prices. Theoretically, a house’s structural characteristics, perceived green space, openness, and perceived footpaths are reflected in house prices [25,46,47,91]. However, in some cases, the ways in which both landscape amenities such as parks and green spaces and the elements that compose the urban environment affect housing prices are not well understood because people have different perceptions and goals [41,65,93–95].

Also, we focused on the “visible” factors of a neighborhood that are visually experienced and the willingness to purchase a house, as well as the price. However, some studies have focused on the hypothesis that people’s cognitive experience of “visible” factors has an important influence on human perception [67,96–98]. This conflicting opinion on the “visible” factors of a neighborhood environment provides room for academic discussion on the relative importance of objective environment and subjective perception in explaining human behavior.

Our analyses confirmed the presence of a non-linear relationship between perceived landscape characteristics and housing prices. Specifically, our interpretable machine-learning model showed that the perceived landscape parameters (perceived green space, perceived pedestrian paths, and enclosure) that correlate with the openness of an urban outdoor space had a non-linear relationship with housing prices. In addition, interaction effects were found between each pair of variables. This finding contrasts with previous research, which showed that visibility from streets and buildings suppressed house prices [25,99–101]. It also contradicts previous research indicating that abundant green spaces in cities increase housing prices [92,102].

Most studies using the HPM have inferred the relationship between housing prices and particular parameters [103–105] because models with good explanatory power are expected to have good predictive power. However, that is not always the case [106–108]. Although explanations can be important, especially for sensitive issues such as house prices, predictive power might be more important. In this study, the predictive power of the gradient boost machine-learning model, an ensemble algorithm, was 86.7%, which represents a surprising 20.7% improvement over the predictive power of the linear regression model. This suggests that machine-learning models can be usefully applied to solve the structural problems with existing statistical models.

The analysis dataset utilized in this research relied on objective indicators: Street View Images, Quantity, Distance, Density-based GIS, and location data. However, objective indicators cannot completely describe people’s sense of a neighborhood environment, as perception is a highly subjective process [6]. In addition, GSV images are considered similar to pedestrians’ visual perspectives because they are captured from vehicles on the road. For this reason, researchers use GSV data to measure streetscape features, which are assumed to be similar to pedestrian perspectives [8,109]. However, it should be noted that strictly speaking, GSV images have some limitations to represent pedestrians’ visual perspectives. This study discussed the interaction between housing prices and neighborhoods only for single-family houses. However, the housing market must consider location factors and market segmentation based on the type of housing [41]. This suggests that further research is needed across different housing types and market segments. Another limitation of our study is the use of Euclidean distance to construct variables. While the Euclidean distance is reasonable for the study’s purpose, utilizing a network distance buffer with detailed road network data could more effectively capture the impact of neighborhood environmental characteristics on housing prices.

Another important finding of this study is the interaction effects between visual landscape characteristics and land use mix. Various tools have been developed to measure land use mix levels, and the entropy index is one of the representative methods for estimating land use mix. However, it should be noted that the land use mix calculation formula based on the entropy index does not classify individual land use characteristics separately. For example, 80% commercial area and 20% residential area have the same land use mix value as 80% residential area and 20% commercial area. This study did not examine the compositional characteristics within the land use mix index. Consequently, further research is required to explore the interactions between compositional aspects of land use mix and streetscape characteristics.

## 6. Conclusion

This study has explored the relationship between streetscapes and the price of single-family houses using big data of single-family house transaction prices in Seoul. To this end, an integrated framework for estimating the price of a single-family house from various perspectives was presented using geography-based big data, deep learning–based computer vision technology, and machine-learning algorithms. Specifically, to quantify how streetscapes are perceived by pedestrians in daily life, we used the GSV image data set, and then we considered the relationship between those streetscapes and the prices of single-family houses close to the street. We found a strong non-linear relationship between the perceived streetscape and the price of a single-family house. Moreover, the interaction between perceived landscape variables and land use mix affected single-family housing prices. Therefore, a differentiated approach is needed for planning and managing streetscapes for considering the non-linear and interactive characteristics of landscape factors in residential neighborhoods.

Meanwhile, since it is difficult to obtain information on single-family housing prices in Korea, most research is focused on apartment prices. However, this study conducted the analysis using credible big data on actual single-family housing transactions obtained through MOU. In particular, while previous studies treated site area, gross floor area, and age as control variables, this study additionally considered site shape, conner parcel, and parking availability. In addition, methodological supplementation was achieved through the combination of deep learning and machine learning, and variables were constructed by scientifically measuring the street environment around a vast number of residential areas based on a deep learning model. In addition, we can confirm the difference from previous studies in that the model’s explanatory power was improved by combining single-family house spatial big data and machine learning models.

This study hypothesized that unconscious visual perception and everyday experiences significantly influence behavior. However, it lacked an in-depth analysis of interactions between physical and perceived environments, risking biased interpretations. Limited tools for measuring homebuyers’ subjective perceptions and data restricted to specific regions and groups further constrained the findings’ accuracy and generalizability. Future studies should include diverse regions and populations to overcome these limitations.

## Supporting information

S1 Data Analytical data set for the Housing_price and Machine learning study. https://doi.org/10.6084/m9.figshare.26965252.
(XLSX)
